# Neuroprotective effect of astrocyte-derived IL-33 in neonatal hypoxic-ischemic brain injury

**DOI:** 10.1186/s12974-020-01932-z

**Published:** 2020-08-28

**Authors:** Mengya Jiao, Xiangyong Li, Liying Chen, Xiaodi Wang, Baohong Yuan, Tao Liu, Qun Dong, Hanfang Mei, Hui Yin

**Affiliations:** 1 Department of Biochemistry and Molecular Biology, Guangdong Pharmaceutical University, Guangzhou, 510006 China; 2 Institute of Biochemistry and Molecular Biology, Guangdong Medical University, Zhanjiang, 524023 China; 3 Guangdong Provincial Key Laboratory of Pharmaceutical Bioactive Substances, Guangdong Pharmaceutical University, Guangzhou, 510006 China; 4 Department of Microbiology and Immunology, Guangdong Pharmaceutical University, Guangzhou, 510006 China

**Keywords:** Neonatal hypoxic-ischemic brain injury, IL-33, ST2, Astrocytes, Neuroprotection

## Abstract

**Background:**

Interleukin-33 (IL-33) is a well-recognized pleiotropic cytokine which plays crucial roles in immune regulation and inflammatory responses. Recent studies suggest that IL-33 and its receptor ST2 are involved in the pathogenesis of neurological diseases. Here, we explore the effect of IL-33/ST2 signaling in neonatal hypoxic-ischemic (HI) brain injury and elucidate the underlying mechanisms of action.

**Methods:**

The brain HI model was established in neonatal C57BL/6 mice by left common carotid artery occlusion with 90 min hypoxia and treated with IL-33 at a dose of 0.2 μg/day i.p. for 3 days. TTC staining and neurobehavioral observation were used to evaluate the HI brain injury. Immunofluorescence and flow cytometry were applied to determine the expression of IL-33 and its receptor ST2 on brain CNS cells and cell proliferation and apoptosis. OGD experiment was used to assay the viability of astrocytes and neurons. RT-qPCR was used to measure the expression of neurotrophic factor-associated genes.

**Results:**

The expression level of IL-33 was markedly enhanced in astrocytes 24 h after cerebral HI in neonatal mice. Exogenous delivery of IL-33 significantly alleviated brain injury 7 days after HI, whereas ST2 deficiency exacerbated brain infarction and neurological deficits post HI. Flow cytometry analyses demonstrated high levels of ST2 expression on astrocytes, and the expression of ST2 was further elevated after HI. Intriguingly, IL-33 treatment apparently improved astrocyte response and attenuated HI-induced astrocyte apoptosis through ST2 signaling pathways. Further in vitro studies revealed that IL-33-activated astrocytes released a series of neurotrophic factors, which are critical for raising neuronal survival against oxygen glucose deprivation.

**Conclusions:**

The activation of IL-33/ST2 signaling in the ischemic brain improves astrocyte response, which in turn affords protection to ischemic neurons in a glial-derived neurotrophic factor-dependent manner.

## Background

Perinatal hypoxic-ischemic encephalopathy (HIE) is a common and devastating disease that is a primary cause of neonatal mortality and long-term neurological deficits in children [1–3]. HIE in the newborn often results from a hypoxic event and subsequent insufficient cerebral blood flow to the brain, leading to millions of neonatal death or long-term disabilities every year [4]. Although there have been major advances in modern technology and an increased understanding of fetal and neonatal pathologies, the therapeutic interventions for HIE in the clinical setting are limited [5, 6]. Thus, it is urgently needed to develop safe and effective therapies for the treatment of neonates suffering HIE.

As a novel member of the IL-1 family of cytokines, IL-33 has been increasingly implicated in a variety of innate and adaptive immune response [7, 8]. IL-33 is constitutively expressed in the nuclei of endothelial and epithelial cells with barrier function, or to be released into the extracellular space, as an alarmin, after tissue damage to alert the immune system [9]. In the cell nucleus, IL-33 may act as a transcriptional repressor and modulate NF-κB-mediated gene expression. The extracellular IL-33 exerts its biological functions by binding to its receptor complex, which is composed of ST2 and IL-1 receptor accessory protein (IL-1RAcP) [10]. It has been recently observed that both IL-33 and its receptor ST2 mRNA are expressed at high levels in the central nervous system (CNS) [11]. Moreover, several studies also reported the involvement of 33/ST2 signaling in the pathogenesis of neurological diseases including Alzheimer’s disease [12], experimental autoimmune encephalomyelitis (EAE) [13], experimental cerebral malaria [14], and stroke [15]. However, the effect of IL-33/ST2 axis in neonatal hypoxic-ischemic brain injury remains unclear.

In the present study, we found that exogenous delivery of IL-33 significantly ameliorated HI-mediated neonatal brain damage, whereas genetic deficiency of ST2 exacerbated brain infarction and behavioral disorders. Further in vivo and in vitro studies showed the importance of the IL-33/ST2 signaling in post-HI astrocyte responses and subsequent protection of ischemic neurons. Mechanistic studies revealed that IL-33/ST2 engagement stimulates the production of neurotrophic factors from astrocytes, which in turn enhances neuronal survival in neonatal HI brain injury.

## Methods

### Mice and IL-33

Seven-day-old (P7) ST2-deficient mouse pups on a C57BL/6 background and their wild-type littermates were obtained from Cyagen Biosciences Inc., Guangzhou, China. All animals were housed in specific pathogen-free conditions, and experiments were approved by the Animal Care and Use Committee of Guangdong Pharmaceutical University. Recombinant mouse IL-33 protein was prepared in our laboratory as described previously [16]. Mice were injected i.p. with IL-33 (0.2 μg per mouse) or PBS (control) for 3 days after hypoxic-ischemic (HI) operation. The dose of IL-33 used in HI neonatal mice was based on the body weight ratio in adult mice with stroke as described previously [15].

### Neonatal hypoxic-ischemic brain injury

The modified Rice-Vannucci model was used to establish hypoxic-ischemic (HI) brain injury [17, 18]. P7 pups were anesthetized with isoflurane and underwent a unilateral left common carotid artery occlusion using bipolar electrical coagulation (Vetroson). The incision was closed using tissue adhesive (3M Vetbond). After a recovery period of 2 h, pups were placed in a hypoxia chamber (8% O_2_, balanced nitrogen) maintained at 37 °C for 90 min. Pups were then recovered and returned to their dams. A subset of control animals were mock-treated with a small incision in their neck without artery occlusion and placed in a chamber at normal air temperature (sham control).

### Infarct volume

Seven days after the termination of hypoxic insult, the brains of pups in HI and sham groups were sliced coronally into 2-mm-thick sections and incubated in 2% 2,3,5-triphenyltetrazolium chloride (TTC) at 37 °C in the dark for 15 min. The areas of ipsilateral and contralateral hemispheres were measured using ImageJ software (National institute of Health, Bethesda, MD, USA). After correcting for edema, the volumes of infarction were calculated as follows: Total volume of contralateral hemisphere − (Total volume of ipsilateral hemisphere − Average volume of 3 measurements of infarct).

### Neurobehavioral evaluation

Pups in each treatment group were subjected to three neurobehavioral tests 1, 3, and 7 days after HI procedure as previously described [19, 20]. (1) Geotaxis reflex is used for diagnosing vestibular and/or proprioceptive functions. Pups were placed with its head downward on a 30° incline, and the latency to make a 90° turn was recorded. (2) Cliff avoidance reaction is used for assessing maladaptive impulsive behavior. Pups were placed on the protruding end of the board with their head down and forepaws off the board, and the latency to place both their forepaws back on the board was measured. (3) Grip test is used for evaluating grip force and fatigability. Pups were suspended by both forepaws on a metallic wire, and the total time to fall was recorded.

### Primary neonatal astrocyte culture

Primary astrocyte cultures were prepared from neonatal mice as described previously [21]. Briefly, cortices from newborn mouse pups were dissected and pooled, and cells dissociated by exposure to of 2.5 % trypsin for 30 min at 37 °C. The cells were centrifuged at 300×*g* for 5 min and resuspended in DMEM containing 10% FBS and 50 U/mL penicillin-streptomycin (Gibco). The cells were plated onto poly-l-lysine coated 25-cm^2^ flasks at a density of 6 × 10^5^ cells/cm^2^ and cultured for 10–12 days. Nonastrocytic cells, such as microglia and neurons, were detached from the flasks by shaking and removed by changing the medium. Astrocytes were dissociated by trypsinization and then reseeded on uncoated 6- and 24-well plates for the following experiments. The purity of GFAP^+^ astrocytes examined by flow cytometry was over 96%.

### Lentivirus transduction

Primary astrocytes were infected with either PUMAα shRNA lentiviral particles (PUMA shRNA LV) or control shRNA LV (Santa Cruz biotechnology) in DMEM with 5 μg/mL polybrene at multiplicities of infection (MOI) of 15. After 12 h of culturing, the medium was replaced by fresh DMEM with 10% FBS in order to remove debris and inactive lentiviruses. Some wells were then supplemented with IL-33 (50 ng/mL) following oxygen-glucose deprivation/reoxygenation (OGD/R) treatment and then collected for cell apoptosis and viability analysis.

### Astrocyte OGD experiment

Primary astrocyte cultures were replaced with the glucose-free DMEM and transferred into an anaerobic chamber flushed with 5% CO_2_ and 95% N_2_ (v/v) at 37 °C for 6 h. The astrocytes were incubated again in DMEM containing 10% FBS and returned to the normoxic incubator (95% air and 5% CO_2_) at 37 °C for 24 h. For IL-33 treatment, cultured astrocytes (5 × 10^5^) were treated with 50 ng/mL IL-33 throughout the whole period of OGD/R (30 h), pretreatment with or without Ly294002 (1 μM; Selleck) for phosphorylation AKT-S473 analysis or P53 inhibitor Pifithrin-α (10 μM; Selleck) for cell apoptosis analysis. Cells without exposure to OGD/R were defined as control group.

### Astrocyte-conditioned media (ACM)

Primary astrocyte cultures were replaced with the glucose-free DMEM and transferred into an anaerobic chamber flushed with 5% CO_2_ and 95% N_2_ at 37 °C for 6 h. Cells were then incubated in neurobasal medium (Gibco) containing 2% B27 (Gibco) and returned to the normoxic conditions for an additional 24 h. Some wells were treated with IL-33 (50 ng/mL) throughout the whole period of OGD/R (30 h). Conditioned media were collected and filtered using a 0.22-μm filter before use in neuron studies.

### Primary cortical neuronal cell culture

Cerebral cortices from newborn mouse pups were dissected and cells dissociated by incubation of OPC papain solution (1.54 mg/mL papain, 360 μg/mL l-cysteine and 60 μg/mL DNase I) for 20 min at 37 °C. Cells were filtered through a 40-μm cell strainer to obtain a single cell suspension, and then resuspended in neurobasal media containing 2% B27. Cells were cultured in a humidifed incubator at 37 °C with 5% CO_2_ for 7 days before experiments.

### Neuron OGD experiment

Primary cultured cortical neurons were then exposed to OGD/R experiments as described previously [22]. Briefly, cell cultures were replaced with the glucose-free DMEM and transferred into an anaerobic chamber flushed with 5% CO_2_ and 95% N_2_ at 37 °C for 3 h. The neurons were incubated again in neurobasal medium containing 2% B27 and returned to the normoxic conditions for an additional 24 h. For IL-33 or astrocyte-conditioned media (ACM) treatment, cultured neurons (5 × 10^5^) were treated with different concentrations (25, 50, 75, and 100 ng/mL) of IL-33 or various doses of ACM throughout the whole period of OGD/R, pretreatment with or without 20 μM of Nintedanib (MedChem Express), a tyrosine kinase inhibitor. Cells without exposure to OGD/R were defined as control group.

### Cell viability

Cell viability was determined by cell counting kit-8 (CCK-8, Dojindo, Japan) assay. Cells were seeded at a density of 1 × 10^3^ cells per well in 96-well plates. After underwent OGD, the cells were cultured with IL-33 or ACM for 24 h and subsequently with CCK-8 solution for 2 h at 37 °C. The absorbance at 450 nm was measured using a microplate reader (Model 680, Bio-Rad Laboratory, Hercules, CA).

### Flow cytometry

To examine the percentages of ST2^+^ cells, cells were suspended in PBS and incubated with PE-labeled anti-ST2 antibody (Biolegend) for 30 min. Cell apoptosis was measured using the Annexin V apoptosis detection kit (eBioscience) and in situ apoptosis detection kit (Roche) according to the manufacturer’s directions. For analysis of phosphorylation AKT-S473, cells were fixed and permeabilized with the BD Cytofix/Cytoperm kit (BD Biosciences) and then stained with mouse anti-phospho-AKT-S473 PE or isotype control (eBioscience). For analysis of RET, PUMA, or p53 protein levels, cells were fixed in 4% paraformaldehyde and permeabilized in 0.25% saponin. Cells were stained with a primary anti-RET, anti-PUMA or anti-p53 antibody (MultiSciences Biotech Co., Ltd, Hangzhou, China) followed by a secondary donkey anti-rabbit PE antibody (Biolegend). Flow cytometric analysis was performed with FACSCalibur cytometer (BD Biosciences) and CellQuest v3.3 software.

### Cell proliferation

For analysis of cell proliferation, cells were cultured with 50 ng/mL IL-33 or ACM following OGD/R (30 h) treatment, fixed and permeabilized using the BD Cytofix/Cytoperm kit, and stained intracellularly with PE anti-Ki-67 (eBioscience). Cell proliferation was analyzed with a FACSCalibur cytometer.

### Cell cycle analysis

Cell were fixed with 75% ethanol at 4 °C for 16 h and then treated with 100 μg/mL ribonuclease A and 50 μg/mL propidium iodide (PI; Sigma, St Louis, MO, USA) at room temperature for 30 min. DNA fluorescence of the stained cells was measured with a FACSCalibur cytometer. The percentages of cells within the G1, S, and G2/M phases of the cell cycle were calculated by the use of ModFit software (Verity, Topsham, ME).

### Immunofluorescence

Mouse pups were anesthetized and transcardially perfused with 4% paraformaldehyde in PBS. The brain tissues were embedded in paraffin and sliced coronally in 5 μm. Tissue sections were dewaxed, quenched with 3% hydrogen peroxide for 10 min, and incubated with 5% bovine serum albumin (BSA) for 30 min. The sections were stained overnight at 4 °C with either anti-ST2 (1:200, ab25877, Abcam), anti-IL-33 (1:500, AF3626, R&D), anti-GFAP (1:500, 104805-T08, Sino Biological Inc.), anti-Olig2 (1:500, EPR2673, Abcam), anti-Iba1(1:50, 20A12.1, Sigma-Aldrich), anti-Neun (1:50, A60, Sigma-Aldrich), or anti-Ki67 (1:500, ab15580, Abcam) followed by PE- or FITC-conjugated secondary antibodies (eBioscience). TUNEL staining was performed with the in situ cell death detection kit (Roche). The slides were counterstained with nuclear dye DAPI and observed on an Olympus BX51 fluorescent microscope.

### Western blotting

Neonatal brains were dissected at the appointed times after HI. Proteins from ipsilateral hemisphere were extracted by tissue homogenization in RIPA buffer containing a proteinase inhibitor cocktail (Santa Cruz). Equal amounts of protein (30 μg/well) were separated by gel electrophoresis and transferred onto polyvinylidene difluoride (PVDF) membranes. Blots were probed with anti-IL-33 (AF3626, R&D), anti-cleaved caspase-3 (9661, Cell Signaling Technology, Inc.), anti-Bax (ab32503, Abcam), and anti-Bcl-2 (ab196495, Abcam) antibodies and then detected using HRP-conjugated secondary antibody (Abcam). β-actin (100166-MM10, Sino Biological Inc., China) was used as a loading control. Images were analyzed using ImageJ and normalized to β-actin.

### Quantitative PCR assay

RNA from primary astrocytes was purified using TRIzol reagent (Invitrogen) and transcribed into cDNA using M-MLV reverse transcriptase (Invitrogen). Quantitative PCR (qPCR) was performed to detect mRNA expression using SYBR Green qPCR kit (Invitrogen) and primers for PCR amplification included: p53 forward 5′-TCA CAG TCG GAT ATC AGC CT-3′, reverse 5′-ACA CTC GGA GGG CTT CAC TT-3′; PUMA forward 5′-CCT CCT TTC TCC GGA GTG TTC A-3′, reverse 5′-ATA CAG CGG AGG GCA TCA GG-3′; GDNF forward 5′-CTC TAG CTC TTG GGG GAA TC-3′, reverse 5′-ACG ACC GAG ACA TCA GAG AG-3′; ARTN forward 5′-TAC TGC ATT GTC CCA CTG CCT CC-3′, reverse 5′-TCG CAG GGT TCT TTC GCT GCA CA-3′; PSPN forward 5′-TGT CAC AAT GGC TGC AGG AAG ACT T-3′, reverse 5′-AGC TCA GCC ACT GGT AGG GTC AGG-3′; NRTN forward 5′-CAG CGG AGG CGC GTG CGC AGA GA-3′, reverse 5′-CGG CTG TGC ACG TCC AGG AAG GA-3′ and GAPDH forward 5′-TTC ACC ACC ATG GAG AAG GC-3′, reverse 5′-GGC ATG GAC TGT GGT CAT GA-3′. All qPCR reactions were performed with an ABI PRISM® 7000 Sequence Detector Systems (Applied Biosystems, Foster City, CA), and expression values were normalized to the housekeeping gene GAPDH using the comparative threshold cycle (CT) method.

### Statistical analyses

Statistical analyses were performed using SPSS version 13.0 (SPSS, Chicago, IL, USA). All the data are presented as the mean ± standard error of the mean (SEM). Statistical differences between groups were evaluated by Student’s *t* test or one way ANOVA. *P* values less than 0.05 were considered significant.

## Results

### HI injury induces an elevation of IL-33 in the neonatal brain

**Fig. 1:**
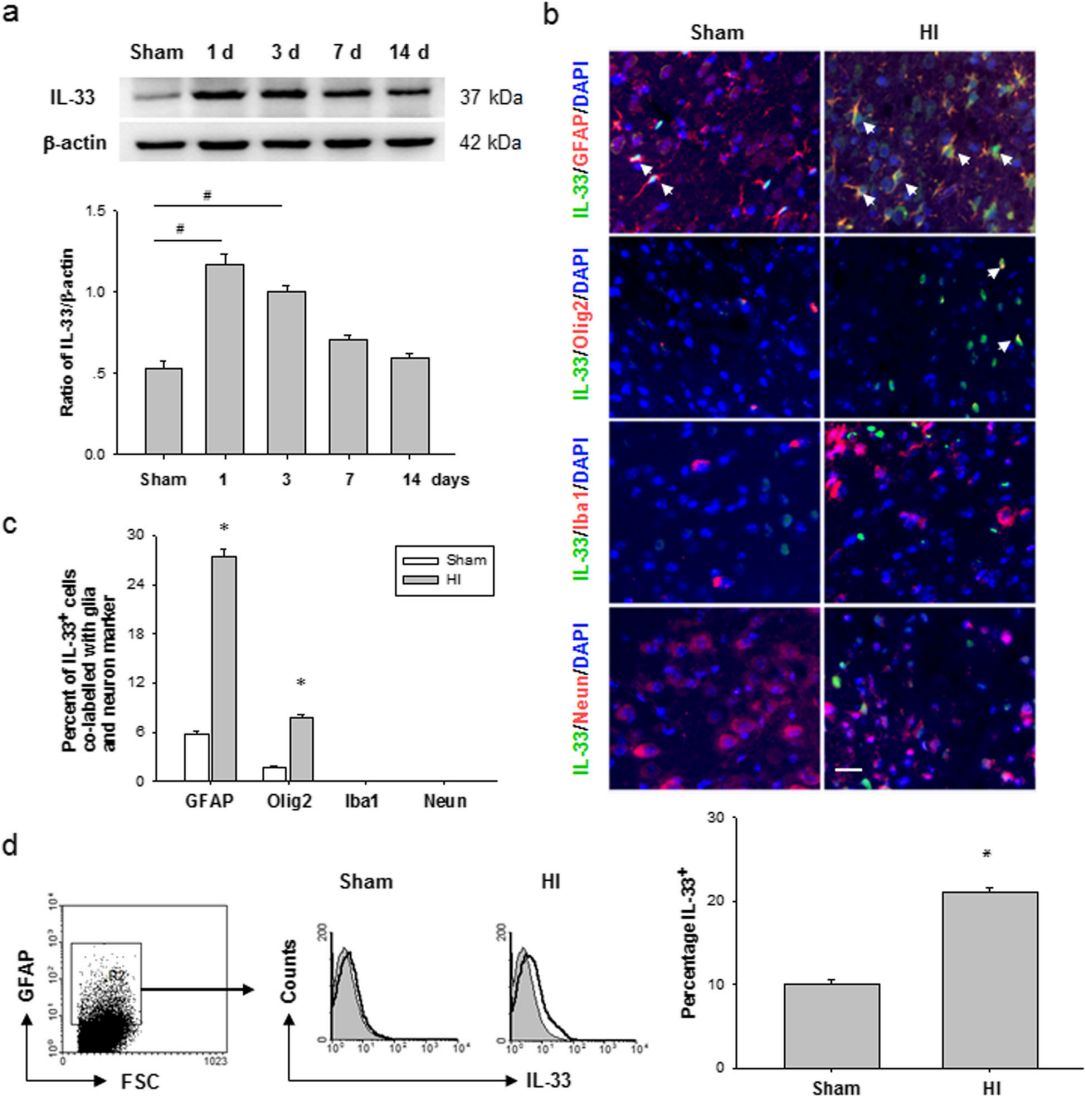
Astrocytic IL-33 is robustly upregulated after hypoxic-ischemia in neonatal mouse brains. **a** Western blot of IL-33 was performed using protein extracted from the mouse brain at 1, 3, 7, and 14 days after HI or sham operation. Data are mean ± SEM (*n* = 6 in each group). ^#^*P* < 0.01 compared to sham. **b** Representative images of IL-33 (green) and GFAP or Olig2 (red) labeling in the cerebral cortex of WT mice at 1 day after HI injury. No IL-33 staining was observed in Neun or Iba1 cells 1 d after HI. Scale bar, 25 μm. Nuclei were stained blue with 4,6-diamidino-2-phenylindole (DAPI). **c** Semi-quantitative analysis of glia or neuron-type cell contributions to the IL-33-positive cell population in vehicle group 1 day after HI. Data are mean ± SEM (*n* = 6 in each group). *P < 0.05 compared to sham. **d** Representative IL-33 expression on astrocytes in WT mice at 1 day after HI injury. The numbers shown indicate the percentage of IL-33 expression on the indicated cell population. Data are mean ± SEM (*n* = 6 in each group). *P < 0.05 compared to sham

To identify the involvement of IL-33 in post-HI neuroprotection, we firstly examined the expression of IL-33 on the brain after HI injury in neonatal mice. A dramatic increase in IL-33 protein was observed at the lesion site 1 day after HI and then decreased in the following days (Fig. 1a). The phenotype of IL-33-expressing cells was identified by immunofluorescent staining, which showed that IL-33 readily detected from astrocytes (IL-33^+^GFAP^+^) in the periinfarct areas, but less or none was present in the microglia and neurons (Fig. 1b, c). Furthermore, flow cytometry analysis confirmed distinctly increased counts of IL-33^+^ astrocytes at 1 day after HI (Fig. 1d). Together, these results suggest that astrocyte-derived IL-33 is a specific factor upregulated after brain HI and that astrocytes are a major source of IL-33 in the CNS.

### IL-33 administration alleviates HI-induced neonatal brain injury

**Fig. 2:**
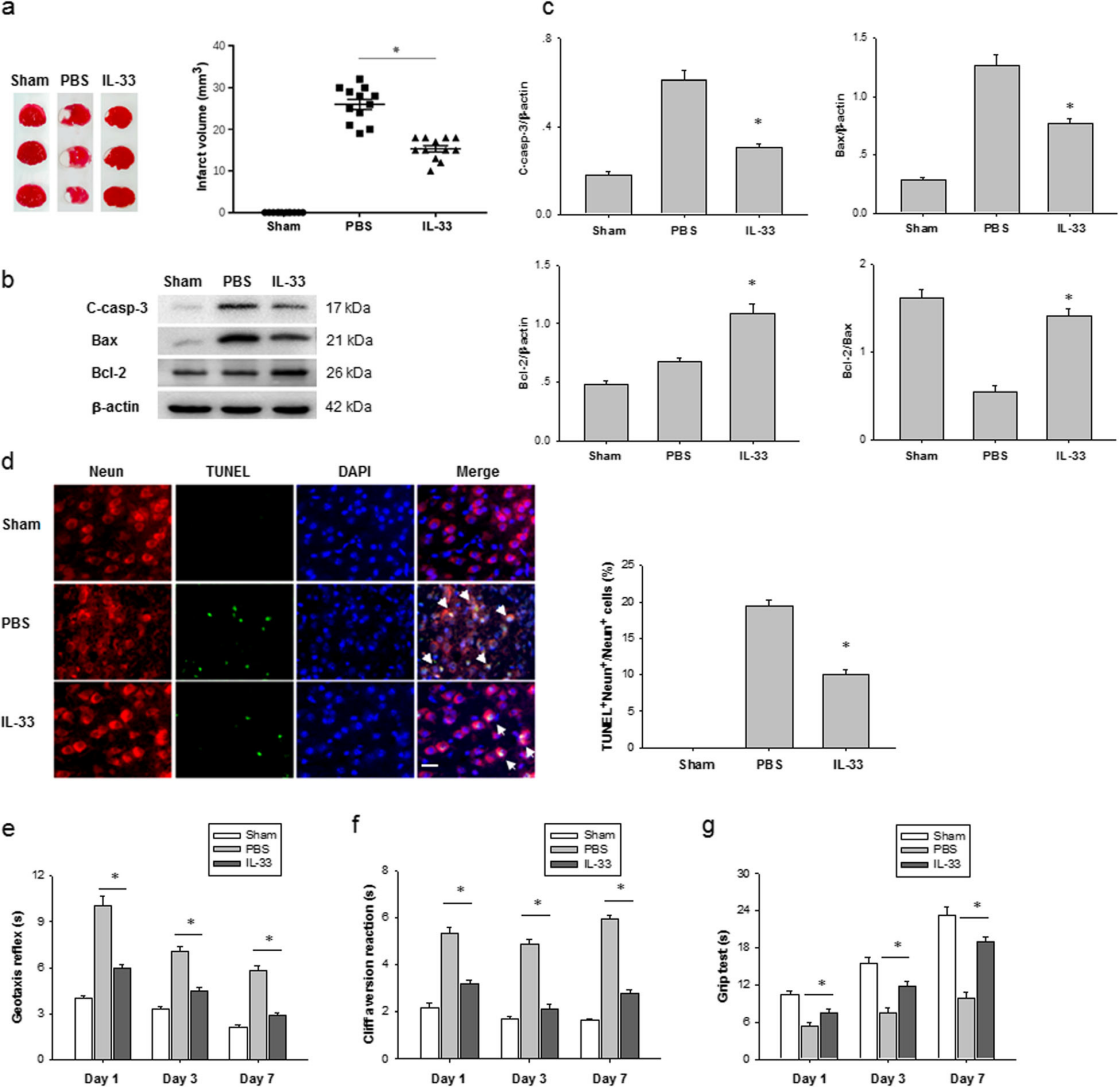
IL-33 ameliorates HI-induced brain injury in neonatal mice. **a** Representative photographs of TTC-stained coronal brain sections from IL-33- and PBS-treated mice at 7 days post HI (left). Right, infarct volumes at 7 days post HI (*n* = 12 per group). **b** Representative western blot images of cleaved caspase-3 (C-casp-3), Bax, and Bcl-2 from IL-33- and PBS-treated mice at 7 days post HI (*n* = 9 per group). **c** Densitometric analysis of data is shown in **b**. **d** Representative images of Neun (red) and TUNEL (green) in the cerebral cortex of IL-33- and PBS-treated mice at 7 days post HI (left). Scale bar, 25 μm. Right, quantification of Neun and TUNEL dual-labeled cells at 7 days post HI (*n* = 9 per group). **e**–**g** Neurobehavioral outcomes of the geotaxis reflex (left), cliff avoidance reaction (middle), and grip test (right) at 1, 3, and 7 days after HI (*n* = 9 per group). Data are mean ± SEM. *P <0.05 compared to untreated controls

Next, we explored whether IL-33 can ameliorate brain damage in vivo using a neonatal mouse HI brain injury model. TTC staining of coronal sections of mouse brains was used for evaluating the infarct volume. We observed that treatment with IL-33 at a dose of 0.2 μg/dose/day for 3 days significantly reduced brain infarct sizes 7 days after HI (Fig. 2a). Exogenous IL-33 markedly reduced the levels of apoptosis-related proteins including cleaved csapases-3 and bax in brain tissue 7 days after HI challenge (Fig. 2b). The protein ratio of Bcl-2/Bax was obviously restored in IL-33-treated group compared to the vehicle-treated group (Fig. 2c). Furthermore, Neun and TUNEL double staining confirmed a significantly decrease in neuronal death (Neun^+^ TUNEL^+^) 7 days after HI in IL-33-treated mice (Fig. 2d).

Neurological functions after HI injury were assessed in sham, vehicle-treated, and IL-33-treated mice. Neurobehavioral tests evaluating the geotaxis reflex, cliff avoidance, and grip were performed 1, 3, and 7 days after HI in the three groups. Compared with pups in the sham group, neurobehavioral deficits of pups in the vehicle-treated HI group were clearly observed 1, 3, and 7 days after HI (Fig. 2e–g). However, IL-33 treatment significantly improved these neurological defects (Fig. 2e–g). Therefore, these data imply that IL-33 not only reduces brain damage but also improves behavioral performance after HI challenge.

### ST2 deficiency exacerbates neonatal brain injury after HI

**Fig. 3:**
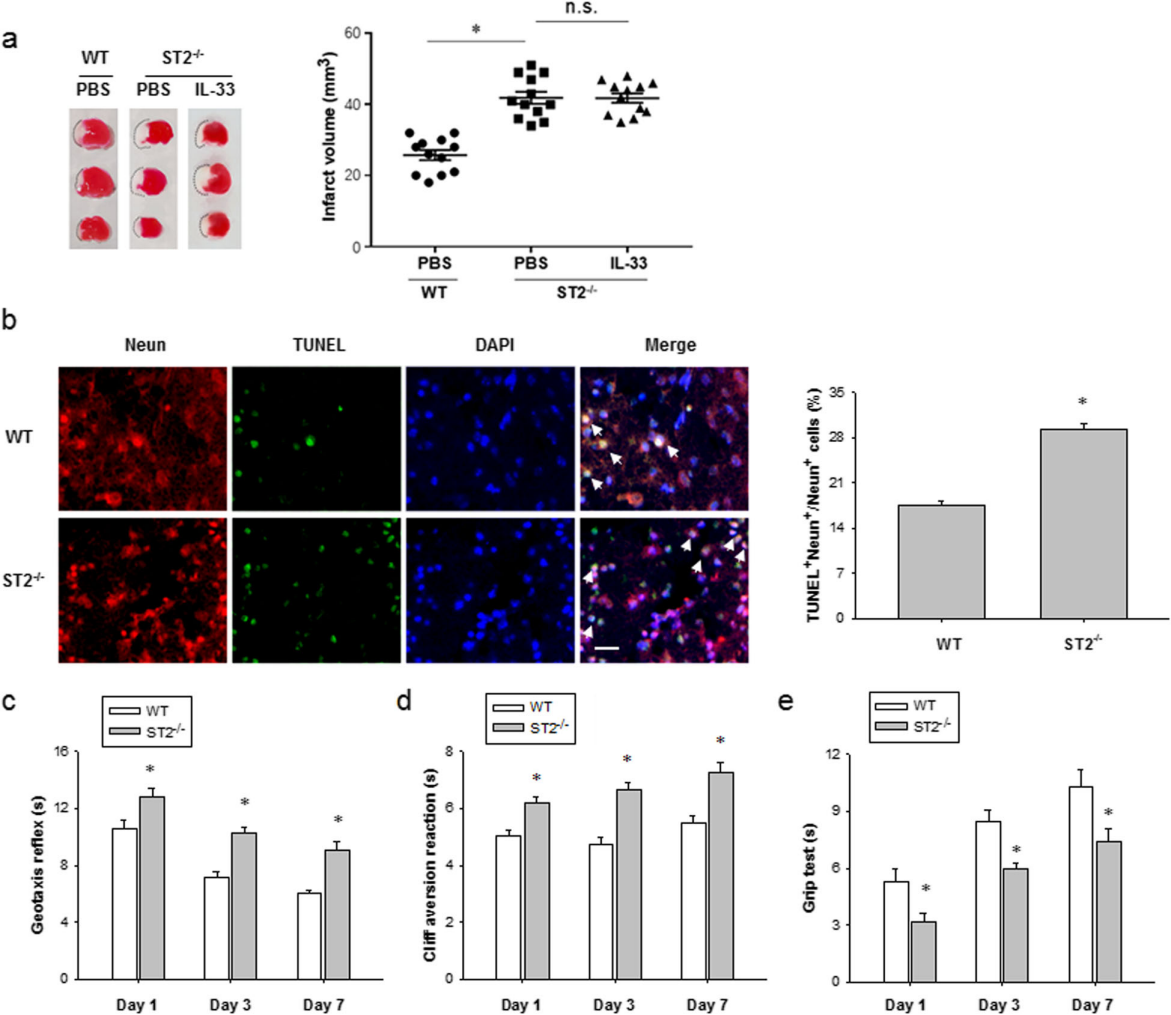
ST2 deficiency aggravates HI-induced brain injury in neonatal mice. **a** TTC staining and infarct volumes in WT and *ST2*^−/−^ mice treated with or without IL-33 at 7 days post HI (*n* = 12 per group). **b** Representative images of Neun (red) and TUNEL (green) in the cerebral cortex of WT and *ST2*^−/−^ mice at 7 days post HI (left). Scale bar, 25 μm. Right, quantification of Neun and TUNEL dual-labeled cells at 7 days post HI (*n* = 9 per group). **c–e** Neurobehavioral outcomes of the geotaxis reflex (left), cliff avoidance reaction (middle), and grip test (right) at 1, 3, and 7 days after HI (*n* = 9 per group). Data are mean ± SEM. *P < 0.05 compared to WT. n.s. not significant

To demonstrate an endogenous role and specificity of IL-33, we performed an equal HI injury in WT and ST2-deficient (*ST2*^*-/-*^) neonatal mice. Infarct volumes were quantified at 7 d after HI using TTC staining. As shown in Fig. 3a, lack of ST2 expression aggravated the brain infarct sizes. Moreover, treatment with IL-33 failed to reduce the ischemic brain damage in neonatal *ST2*^*−/−*^ mice. *ST2*^−/−^ mice displayed significantly increased cerebral accumulations of TUNEL^+^ Neun^+^ cells after HI compared with their WT littermates (Fig. 3b). Meantime, sensorimotor tests such as the geotaxis reflex (Fig. 3c), cliff avoidance (Fig. 3d), and grip (Fig. 3e) consistently revealed worse neurological performance in *ST2*^−/−^ mice up to 7 days after HI. The sham WT and sham *ST2*^−/−^ mice exhibited no significant differences in any behavioral tests, suggesting equivalent baselines in both genotypes (data not shown). Together, these data indicate that the receptor ST2 is crucial for IL-33 exerting the neuroprotective effect after brain HI injury.

### ST2 is highly expressed on astrocytes under physiological and pathological conditions

**Fig. 4:**
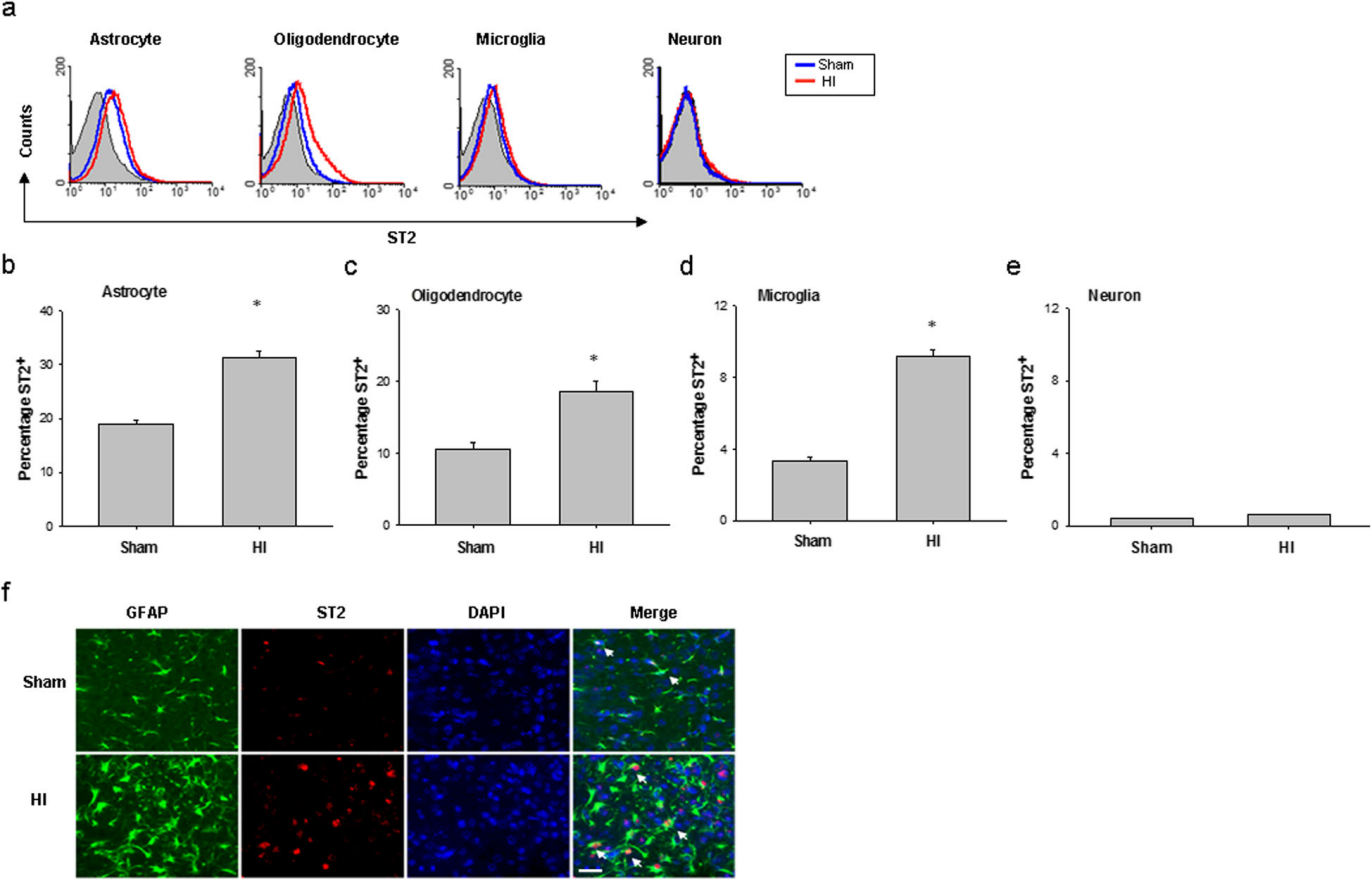
ST2 expression on astrocytes is elevated after HI. **a** Histograms showing ST2 expression on astrocytes, oligodendrocytes, microglia, and neurons in the brain 3 days after HI or sham operation. The blue and red lines represent staining in sham and HI brains, respectively, at 3 days after surgery. The gray area represents isotype control staining. **b–e** Percentages of ST2 expression on astrocytes (**b**), oligodendrocytes (**c**), microglia (**d**), and neurons (**e**) in sham brain and HI brain at 3 days after surgery. Data are mean ± SEM (*n* = 5–7 per group), *P < 0.05 compared to sham. **f** Representative images of GFAP (green) and ST2 (red) labeling in the cerebral cortex 3 days after HI. Scale bar, 25 μm. Nuclei were stained blue with DAPI

To determine the cellular targets of IL-33/ST2 signaling in the brain, we used flow cytometry to analyze the expression of the ST2 receptor on different CNS cells in the normal and the ischemic brain at 3 days after HI induction. In normal brains, ST2 was mainly expressed in GFAP^+^ astrocytes. After brain HI, the expression of ST2 was dramatically increased in this population (Fig. 4a–e). Immunohistochemical staining confirmed the expression of ST2 on GFAP^+^ astrocytes 3 days after HI (Fig. 4f). There was no expression of ST2 in neurons before or after HI (Fig. 4). Together, the data gathered thus far suggest that HI increases the expression of the ST2 receptor on brain astrocytes, which may modulate the astrocyte activity in an autocrine manner during HI injury.

### IL-33 enhances astrocyte survival and proliferation after HI injury

**Fig. 5:**
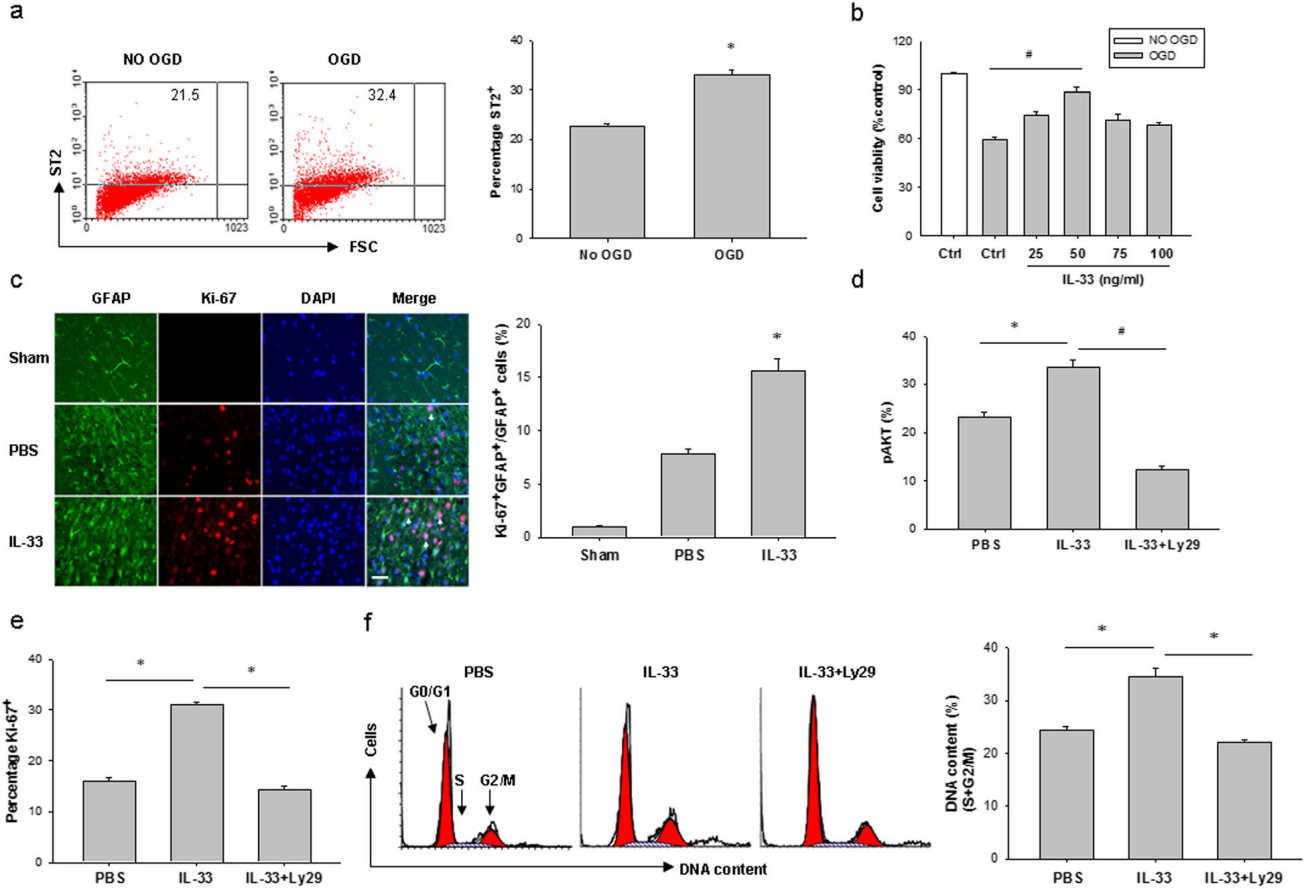
IL-33 promotes astrocyte survival and proliferation after HI injury. **a** Representative FACS analysis of ST2^+^ astrocytes at 24 h after 6 h OGD. Right, numbers indicate the percentage of ST2^+^ astrocytes at 24 h after 6 h OGD. Data are mean ± SEM (*n* = 3 in each group). *P < 0.05 compared to untreated controls. **b** CCK-8 assay in astrocyte-enriched cultures subjected to 6 h OGD or sham conditions followed by treatment with PBS or a range of concentrations of IL-33 for another 24 h. Data are mean ± SEM (*n* = 3 in each group). ^#^*P* < 0.01 compared to untreated controls. **c** Representative images of Ki-67 (red) and GFAP (green) in the cerebral cortex of IL-33- and PBS-treated mice at 7 days post HI (left). Scale bar, 25 μm. Right, quantification of Ki-67 and GFAP dual-labeled cells at 7 days post HI (*n* = 9 per group). **d**, **e** Percentages of phosphorylated AKT (pAKT) (**d**) and Ki-67^+^ (**e**) in astrocytes at 24 h after 6 h OGD and the culture conditions shown. Data are mean ± SEM (*n* = 3 in each group). *P < 0.05, ^#^*P* < 0.01. **f** Representative FACS analysis of cell-cycle status of astrocytes at 24 h after 6 h OGD and the culture conditions shown. Data are mean ± SEM (*n* = 3 in each group). *P < 0.05

As astrocytes express high levels of ST2 under normal conditions and after HI (Fig. 4), we assessed the direct effects of IL-33 on astrocyte survival in vitro. Primary astrocytes were subjected to 6 h oxygen-glucose deprivation (OGD) and treated with different concentrations of IL-33 or vehicle (PBS). Flow cytometry analysis demonstrated high expression of ST2 on astrocytes, which was further upregulated after OGD for another 24 h culture (Fig. 5a). Astrocyte survival was measured 24 h later using CCK-8 assay. There was a dramatic decrease in astrocyte viability after OGD insult, which was significantly rescued by IL-33 after treatment at concentrations ranging from 25 to 100 ng/mL (Fig. 5b). The maximal efficacy of IL-33 was achieved at 50 ng/mL, which was used for all subsequent in vitro experiments.

PI3K/Akt pathway is known as to be the pro-survival and proliferation cell signaling. Here, we asked whether IL-33-mediated astrocyte protection involved PI3K/Akt pathway. IL-33 administration of mice for 3 days after brain HI obviously increased counts of Ki-67^+^ astrocytes in the periinfarct areas compared to PBS-treated HI mice (Fig. 5c). In OGD astrocytes, IL-33 treatment increased AKT phosphorylation corresponded with elevated cell proliferation (Fig. 5d, e). Treatment of OGD astrocytes with Ly294002, a PI3K inhibitor, blocked IL-33-mediated AKT phosphorylation and inhibited cell proliferation in response to IL-33 (Fig. 5d, e). OGD astrocytes treated with IL-33 and Ly294002 also showed apparently reduced cell cycling compared to astrocytes treated with IL-33 alone (Fig. 5f). All together, these data suggest that IL-33 treatment facilitates astrocyte cycling and cell proliferation, which is related to, at least in part, activation of the PI3K-AKT pathway.

### IL-33 inhibits astrocyte apoptosis by repression of PUMA

**Fig. 6:**
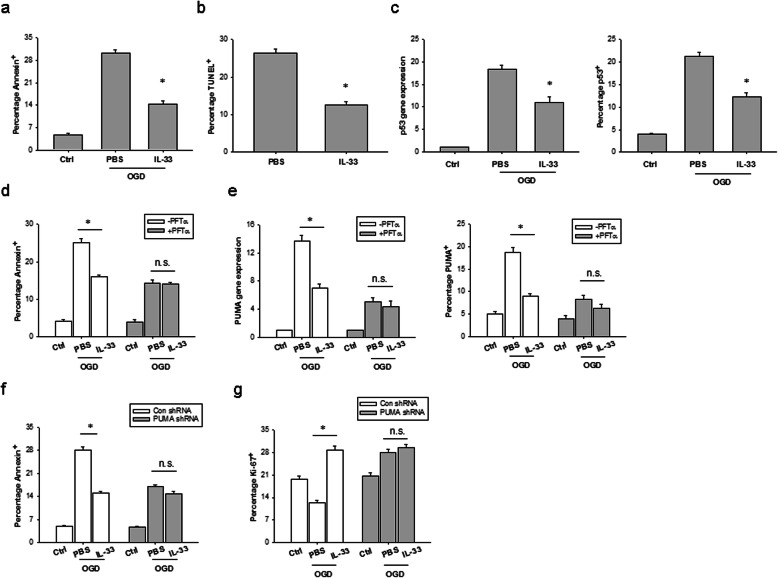
IL-33 protects astrocytes from apoptosis after OGD insult. **a** The percentage of annexin^+^ astrocytes at 24 h of culture with or without IL-33 and after 6 h OGD and the culture conditions shown. Data are mean ± SEM (*n* = 3 in each group). *P < 0.05 compared to untreated controls. **b** The percentage of TUNEL^+^ astrocytes from IL-33- and PBS-treated mice at 7 days post HI. Data are mean ± SEM (*n* = 6 in each group). *P < 0.05 compared to untreated controls. **c** Left, P53 mRNA expression in astrocytes at 12 h of culture with or without IL-33 and after 6 h OGD and the culture conditions shown. Right, mean percentages of P53 protein expression in astrocytes at 24 h of culture with or without IL-33 and after 6 h OGD and the culture conditions shown. Data are mean ± SEM (*n* = 3 in each group). *P < 0.05 compared to untreated controls. **d** The percentage of annexin^+^ astrocytes with or without P53 inhibition at 24 h of culture with or without IL-33 and after 6 h OGD and the culture conditions shown. Data are mean ± SEM (*n* = 3 in each group). *P < 0.05 compared to untreated controls. n.s. not significant. **e** Left, PUMA mRNA expression in astrocytes at 12 h of culture with or without IL-33 and after 6 h OGD and the culture conditions shown. Right, mean percentages of PUMA protein expression in astrocytes at 24 h of culture with or without IL-33 and after 6 h OGD and the culture conditions shown. Data are mean ± SEM (*n* = 3 in each group). *P < 0.05 compared to untreated controls. n.s. not significant. **f** The percentage of annexin^+^ astrocytes with or without PUMA knockdown at 24 h of culture with or without IL-33 and after 6 h OGD and the culture conditions shown. Data are mean ± SEM (*n* = 3 in each group). *P < 0.05 compared to untreated controls. n.s. not significant. **g** Ki-67 assay in astrocytes with or without PUMA knockdown at 24 h of culture with or without IL-33 and after 6 h OGD and the culture conditions shown. Data are mean ± SEM (*n* = 3 in each group). *P <0.05 compared to untreated controls. n.s. not significant

We further investigated whether the beneficial effect of IL-33 on astrocyte survival is associated with inhibition of cell apoptosis after HI injury. At 24 h after OGD challenge, IL-33-treated cultures contained twofold decreased numbers of annexin-positive astrocytes compared to vehicle cultures (Fig. 6a). C57BL/6 mice subjected to brain HI and then treated with IL-33 for 3 days exhibited more than twofold decreased numbers of TUNEL-positive astrocytes compared to PBS-treated controls (Fig. 6b).

Growing evidence showed that PUMA is involved in ischemia/reperfusion-induced cerebral astrocyte apoptosis [23]. Because IL-33 improved astrocyte survival after HI, we explored whether IL-33 causes such effects through repression of PUMA. An increase in p53 mRNA and protein was observed in astrocytes subjected to OGD, which was markedly decreased by IL-33 treatment (Fig. 6c). IL-33 repressed OGD-induced astrocyte apoptosis but had no effect on those pretreated with Pifithrin-α (PFTα), a p53 inhibitor (Fig. 6d). PUMA expression elevated remarkably in astrocytes subjected to OGD, where PUMA mRNA and protein expression did not change in PFTα-pretreated OGD astrocytes, indicating that PUMA induction in astrocytes is p53 dependent (Fig. 6e). IL-33 treatment inhibited OGD-induced PUMA expression in astrocytes but had no effect on PFTα-pretreated astrocytes (Fig. 6e).

Primary astrocytes with specific PUMAα knockdown showed a lower percentage of apoptotic cells and increased cell survival at 24 h after OGD compared to PUMA-expressing astrocytes (Fig. 6f, g). IL-33 treatment increased astrocyte survival and proliferation in PUMA-expressing astrocyte cultures after OGD but had no effect on astrocyte survival or proliferation in PUMA deficient astrocyte cultures after OGD (Fig. 6f, g). All together, these data suggest that IL-33 treatment inhibits OGD-induced astrocyte apoptosis, which is dependent on suppression of PUMA.

### ST2 signaling in astrocytes is essential for their neuroprotective effect in vitro

**Fig. 7:**
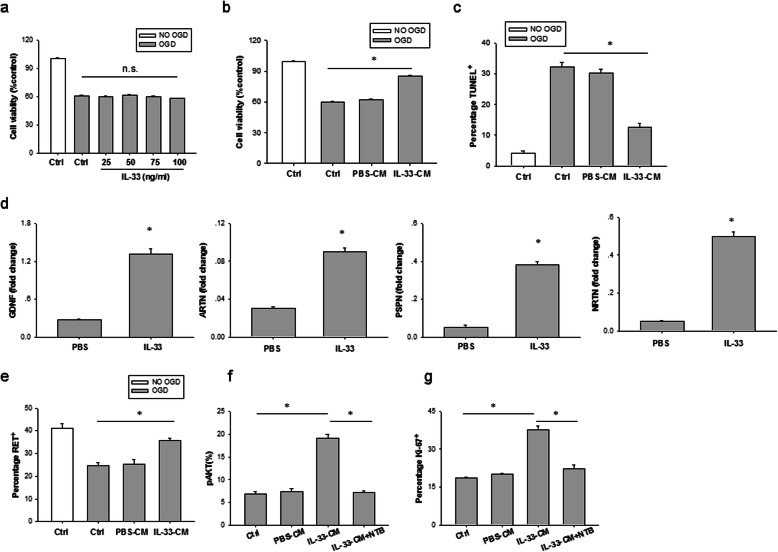
ST2 signaling in astrocytes is essential for their neuroprotective effects in vitro. **a** CCK-8 assay in neuron-enriched cultures subjected to 3 h OGD or sham conditions followed by treatment with PBS or a range of concentrations of IL-33 for another 24 h. Data are mean ± SEM (*n* = 3 in each group). n.s. not significant. **b**, **c** CCK-8 assay (**b**) and TUNEL measure (**c**) in neurons at 24 h of culture with CM from untreated OGD astrocytes (PBS-CM) or CM from IL-33-treated OGD astrocytes (IL-33-CM) and after 3 h OGD and the culture conditions shown. Data are mean ± SEM (*n* = 3 in each group). *P < 0.05 compared to untreated controls. **d** mRNA expression of glial-derived neurotrophic factors GDNF, ARTN, PSPN, and NRTN in astrocytes at 24 h of culture with or without IL-33 and after 6 h OGD and the culture conditions shown. Data are mean ± SEM (*n* = 3 in each group). *P < 0.05 compared to untreated controls. **e** Mean percentages of RET protein expression in neurons at 24 h of culture with PBS-CM or IL-33-CM and after 3 h OGD and the culture conditions shown. Data are mean ± SEM (*n* = 3 in each group). *P < 0.05 compared to untreated controls. **f**, **g** Percentages of phosphorylated AKT (pAKT) (**f**) and Ki67^+^ (**g**) in neurons with or without RET inhibition at 24 h of culture with PBS-CM or IL-33-CM and after 3 h OGD and the culture conditions shown. Data are mean ± SEM (*n* = 3 in each group). *P < 0.05

Finally, we used neuron cultures to explore the potential mechanisms of IL-33-afforded neuroprotection. Primary neurons were subjected to 3 h OGD and then treated with different concentrations of IL-33 or vehicle (PBS). As shown in Fig. 7a, neuronal viability, as measured by the CCK-8 assay, was significantly decreased 24 h after OGD challenge. However, treatment with IL-33 offered no protection to OGD neurons compared with vehicle treatment.

We sought to investigate whether activation of IL-33/ST2 signaling in astrocytes preserves ischemic neurons in vitro. Astrocytes were subjected to 6 h OGD in the presence or absence of IL-33, and then conditioned media (CM) from OGD astrocytes were collected after 24 h normoxic culture. These CM were added to the neuron-enriched cultures that had been exposed to 3 h OGD. Intriguingly, treatment with IL-33-CM obviously enhanced neuronal survival and abrogated neuronal apoptosis when compared with PBS-CM treatment 24 h after OGD challenge, as demonstrated by CCK-8 and TUNEL assay (Fig. 7b, c).

As neurotrophic factors are essential environmental cues to neuronal survival [24], we here assessed whether neurotrophic factors are critical protective mediators released by IL-33-treated astrocytes. The expression of glial-derived neurotrophic factor family ligands (GFL) such as GDNF, ARTN, PSPN, and NRTN were measured by real-time PCR. There was a decrease in the levels of astrocyte GFL after 6 h OGD, which was significantly upregulated after IL-33 treatment (Fig. 7d). C57BL/6 mice subjected to brain HI and then treated with IL-33 for 3 days exhibited higher expression of GDNF and PSPN mRNA than that in PBS-treated controls (Supplementary Fig. S1). Moreover, flow cytometry showed that IL-33-CM reversed the decreased expression of the GFL receptor, namely, the tyrosine kinase receptor RET, on neurons caused by 3 h OGD challenge (Fig. 7e). Treatment with IL-33-CM apparently increased neuronal survival and proliferation in RET-expressing neuron cultures after OGD but had no effect on neuronal survival or proliferation in RET-inhibited neurons after OGD challenge (Fig. 7f, g). Together, these studies strongly suggest that activation of the IL-33/ST2 axis in astrocytes promotes the production of neurotrophic factors, which in turn protect neurons from ischemic injury.

## Discussion

There is a series of studies that IL-33 is released from CNS cells quickly after damage and subsequently triggers the activation of immune responses in lesion areas [25, 26]. In line with these observations, the current study identified that the levels of IL-33 were rapidly elevated in the lesion site 1 day after neonatal HI brain injury. And enhanced IL-33 expression was predominantly localized to astrocytes after HI insult. These findings prompted us to explore the effect of the IL-33/ST2 signaling in neonatal HI brain injury. We found that systemic delivery of IL-33 remarkably alleviated HI-mediated cerebral damage, whereas loss of ST2 aggravated brain infarction and neurological deficits. The activation of IL-33/ST2 signaling in the ischemic brain improved astrocyte response, which in turn afforded protection to ischemic neurons in a glial-derived neurotrophic factor-dependent manner.

Astrocytes are the most abundant type of glial cell within the mammalian brain, which activity is crucial for neuroprotection during brain ischemic damage [27]. Increment studies show that ischemia can induce cell cycle arrest or apoptotic cell death of cortical astrocytes in infarct area [28]. Ischemia-induced growth arrest of astrocytes can be overridden by inhibition of regulator of G protein signaling 2 (RGS2) and treatment with cytokine erythropoietin (EPO) [28, 29], and cytokine-mediated induction of astrocyte survival early post-ischemia may contribute to neuroprotective effect [30]. Although the precise mechanism behind these effects remains unclear, these treatments may induce synchronous entry of cells into the late S phase that is associated with apoptotic resistance [28, 31]. In nonhematopoietic tissues, IL-33 receptor ST2 has been reported to modulate cell proliferation through activation of the PI3K-AKT pathway [32]. Consistent with these findings, we here observe that cerebral astrocytes express high levels of ST2 under normal conditions and even higher levels upon HI challenge. Exogenous administration of IL-33 apparently improves astrocyte survival and proliferation after HI damage. Interestingly, IL-33 attenuates HI-induced growth arrest by promoting astrocyte cycling and that this effect is dependent on activation of the PI3K-AKT signaling. These results suggest that IL-33-mediated upregulation of astrocyte viability provides beneficial neuroprotection against neonatal HI injury.

The p53 upregulated modulator of apoptosis (PUMA) was discovered as a transcriptional target of p53 and exerted a powerful apoptosis-promoting activity [33]. PUMA activates the mitochondrial pathway via the Bcl-2 family member Bax/Bak following neutralizing all members of antiapoptotic Bcl-2 like molecules [34]. Recent studies show that blocking of PUMA signaling protects astrocytes against ischemic-caused death and may confer a significant neuroprotection to brain ischemic/reperfusion injury [35]. In this study, we demonstrate that PUMA is a powerful executor of p53-mediated apoptosis in cerebral astrocytes after HI insult. IL-33 treatment represses HI-induced upregulation of PUMA and downstream apoptotic protein in reactive astrocytes. Furthermore, the effects of IL-33, mediating apoptotic resistance in astrocytes, are dependent largely on repression of PUMA transcription. These findings are consistent with the recently published results showing that inhibition of aberrant p53-PUMA feedback loop activation by treatment with PUMA-specific siRNA and inhibitor pifithrin-α prevented ischemia reperfusion-induced neuroapoptosis and inflammatory responses [36].

It has become increasingly evident that astrocytes play a crucial role in neuron survival during brain ischemia/reperfusion injury [37]. Ischemic lesion in the brain stimulates enhanced expression and release of neurotrophic factors from reactive astrocytes supplying the neurons with appropriate mediators necessary for their survival and maintenance [38]. Therefore, it is critical to determine the specific effectors or functions of the IL-33-stimulated astrocyte response. In line with this goal, our mechanistic studies have identified the glial-derived neurotrophic factor (GDNF) family ligands (GFL) as key molecules released by IL-33-stimulated astrocytes and the major neuroprotective factors in the ischemic brain. The conditioned media derived from IL-33-stimulated astrocytes significantly activate the GFL receptor, a tyrosine kinase receptor RET, and protect neuron survival against ischemic insult. On the contrary, inhibition of the RET receptor almost completely abrogated the protective effects of IL-33 in ischemic neuron culture. In agreement with these data, recent studies demonstrated that neurotrophic factors play a critical role in the survival, differentiation, and maintenance of neurons [39]. Exogenous delivery of neurotrophic factor artemin can have protective effects in animal models of experimental nerve injury, as measured by increased regeneration and functional recovery, and transient reductions in hyperalgesia following nerve injury [40, 41]. Therefore, the capacity of IL-33 to induce neurotrophic factor production in astrocytes may serve as an important mediator of IL-33-afforded CNS protection.

Interestingly, recent studies have reported the immunoregulatory effects of IL-33 in a mouse model of ischemic stroke. Exogenous IL-33 administration decreases stroke-induced CNS damage and ameliorates neurological deficits via increasing anti-inflammatory responses systemically and M2-type macrophages and microglia in the CNS [15]. In addition, IL-33 pretreatment protects mice from ischemic injury by inducing a shift from Th1 to Th2 and suppressing Th17 responses [42], or by potentiating the expression of IL-10 and other M2 genes in microglia [43]. Here, we also found that infusion of IL-33 promotes the development of M2 microglia and macrophages after HI in neonatal mice (data not shown). Furthermore, the potential role of IL-33-polarized M2 microglia and macrophages in neonatal HI mice is currently being addressed.

## Conclusions

The current study reveals a previously unknown function of IL-33/ST2 signaling in neuroprotective effect during neonatal HI brain injury. The activation of IL-33/ST2 axis in the ischemic brain improves astrocyte response, which in turn affords protection to ischemic neurons in a neurotrophic factor-dependent manner. Translationally, these findings imply that IL-33/ST2 signaling may represent a potent new immunotherapy for neonates with hypoxic ischemic encephalopathy.

## Supplementary information

**Additional file 1: Supplementary Fig. S1.** mRNA expression of neurotrophic factors GDNF and PSPN in HI brain tissue of neonatal mice with or without IL-33 treatment for 3 days. Data are mean ± SEM (*n* = 3 in each group). *P < 0.05 compared to untreated controls

## Data Availability

The authors declare that all the data supporting the findings of this study are available within the article and that no data sharing is applicable to this article.

## Abbreviations

IL-33: Interleukin-33
CNS: Central nervous system
HIE: Hypoxic-ischemic encephalopathy
TTC: 2,3,5-Triphenyltetrazolium chloride
OGD/R: Oxygen-glucose deprivation/reoxygenation
PUMA: p53 upregulated modulator of apoptosis
RT-qPCR: Real-time quantitative PCR
MOI: Multiplicities of infection
ACM: Astrocyte-conditioned media
IL-1RAcP: IL-1 receptor accessory protein
GFL: Glial-derived neurotrophic factor family ligands
RGS2: Regulator of G protein signaling 2
EPO: Erythropoietin
GDNF: Glial-derived neurotrophic factor

## References

[1] Hagberg H, Mallard C, Ferriero DM, Vannucci SJ, Levison SW, Vexler ZS (2015). The role of inflammation in perinatal brain injury. Nat Rev Neurol.

[2] Yıldız EP, Ekici B, Tatlı B (2017). Neonatal hypoxic ischemic encephalopathy: an update on disease pathogenesis and treatment. Expert Rev Neurother.

[3] Wachtel EV, Verma S, Mally PV. Update on the current management of newborns with neonatal encephalopathy. Curr Probl Pediatr Adolesc Health Care. 2019;100636. 10.1016/j.cppeds.2019.07.001 31371100

[4] Thornton C, Rousset CI, Kichev A, Miyakuni Y, Vontell R, Baburamani AA (2012). Molecular mechanisms of neonatal brain injury. Neurol Res Int.

[5] Xiong T, Qu Y, Mu D, Ferriero D (2011). Erythropoietin for neonatal brain injury: opportunity and challenge. Int J Dev Neurosci.

[6] Nair J, Kumar VHS (2018). Current and emerging therapies in the management of hypoxic ischemic encephalopathy in neonates. Children (Basel).

[7] Schmitz J, Owyang A, Oldham E, Song Y, Murphy E, McClanahan TK (2005). IL-33, an interleukin-1-like cytokine that signals via the IL-1 receptor-related protein ST2 and induces T helper type 2-associated cytokines. Immunity..

[8] Liew FY, Pitman NI, McInnes IB (2010). Disease-associated functions of IL-33: the new kid in the IL-1 family. Nat. Rev. Immunol..

[9] Liew FY, Girard JP, Turnquist HR (2016). Interleukin-33 in health and disease. Nat Rev Immunol.

[10] De la Fuente M, MacDonald TT, Hermoso MA (2015). The IL-33/ST2 axis: role in health and disease. Cytokine Growth Factor Rev.

[11] Fairlie-Clarke K, Barbour M, Wilson C, Hridi SU, Allan D, Jiang HR (2018). Expression and function of IL-33/ST2 axis in the central nervous system under normal and diseased conditions. Front Immunol.

[12] Fu AK, Hung KW, Yuen MY, Zhou X, Mak DS, Chan IC (2016). IL-33 ameliorates Alzheimer’s disease-like pathology and cognitive decline. Proc Natl Acad Sci U S A.

[13] Russi AE, Ebel ME, Yang Y, Brown MA (2018). Male-specific IL-33 expression regulates sex-dimorphic EAE susceptibility. Proc Natl Acad Sci U S A.

[14] Strangward P, Haley MJ, Albornoz MG, Barrington J, Shaw T, Dookie R (2018). Targeting the IL33-NLRP3 axis improves therapy for experimental cerebral malaria. Proc Natl Acad Sci U S A.

[15] Korhonen P, Kanninen KM, Lehtonen Š, Lemarchant S, Puttonen KA, Oksanen M (2015). Immunomodulation by interleukin-33 is protective in stroke through modulation of inflammation. Brain Behav Immun.

[16] Yin H, Li X, Hu S, Liu T, Yuan B, Gu H (2013). IL-33 accelerates cutaneous wound healing involved in upregulation of alternatively activated macrophages. Mol Immunol.

[17] Rice JE, Vannucci RC, Brierley JB (1981). The influence of immaturity on hypoxic-ischemic brain damage in the rat. Ann Neurol.

[18] Levine S (1960). Anoxic-ischemic encephalopathy in rats. Am J Pathol.

[19] Xu B, Xiao AJ, Chen W, Turlova E, Liu R, Barszczyk A (2016). Neuroprotective effects of a PSD-95 inhibitor in neonatal hypoxic-ischemic brain injury. Mol Neurobiol.

[20] Xiao AJ, Chen W, Xu B, Liu R, Turlova E, Barszczyk A (2015). Marine compound xyloketal B reduces neonatal hypoxic-ischemic brain injury. Mar Drugs.

[21] Schildge S, Bohrer C, Beck K, Schachtrup C. Isolation and culture of mouse cortical astrocytes. J Vis Exp. 2013;71. 10.3791/50079 PMC3582677 23380713

[22] Sun HS, Jackson MF, Martin LJ, Jansen K, Teves L, Cui H (2009). Suppression of hippocampal TRPM7 protein prevents delayed neuronal death in brain ischemia. Nat Neurosci.

[23] Ouyang YB, Xu L, Lu Y, Sun X, Yue S, Xiong XX (2013). Astrocyte-enriched miR-29a targets PUMA and reduces neuronal vulnerability to forebrain ischemia. Glia..

[24] Barde YA (1989). Trophic factors and neuronal survival. Neuron..

[25] Gadani SP, Walsh JT, Smirnov I, Zheng J, Kipnis J (2015). The glia-derived alarmin IL-33 orchestrates the immune response and promotes recovery following CNS injury. Neuron..

[26] Pomeshchik Y, Kidin I, Korhonen P, Savchenko E, Jaronen M, Lehtonen S (2015). Interleukin-33 treatment reduces secondary injury and improves functional recovery after contusion spinal cord injury. Brain Behav Immun.

[27] Barreto G, White RE, Ouyang Y, Xu L, Giffard RG (2011). Astrocytes: targets for neuroprotection in stroke. Cent Nerv Syst Agents Med Chem.

[28] Endale M, Kim SD, Lee WM, Kim S, Suk K, Cho JY (2010). Ischemia induces regulator of G protein signaling 2 (RGS2) protein upregulation and enhances apoptosis in astrocytes. Am J Physiol Cell Physiol.

[29] Jeong JE, Park JH, Kim CS, Lee SL, Chung HL, Kim WT (2017). Neuroprotective effects of erythropoietin against hypoxic injury via modulation of the mitogen-activated protein kinase pathway and apoptosis. Korean J Pediatr.

[30] He ML, Lv ZY, Shi X, Yang T, Zhang Y, Li TY (2017). Interleukin-10 release from astrocytes suppresses neuronal apoptosis via the TLR2/NFκB pathway in a neonatal rat model of hypoxic-ischemic brain damage. J Neurochem.

[31] Wang Y, Yao M, Zhou C, Dong D, Jiang Y, Wei G (2010). Erythropoietin promotes spinal cord-derived neural progenitor cell proliferation by regulating cell cycle. Neuroscience..

[32] Han L, Zhang M, Liang X, Jia X, Jia J, Zhao M (2017). Interleukin-33 promotes inflammation-induced lymphangiogenesis via ST2/TRAF6-mediated Akt/eNOS/NO signalling pathway. Sci Rep.

[33] Wu WS, Heinrichs S, Xu D, Garrison SP, Zambetti GP, Adams JM (2005). Slug antagonizes p53-mediated apoptosis of hematopoietic progenitors by repressing puma. Cell..

[34] Vávrová J, Rezáčová M (2014). Importance of proapoptotic protein PUMA in cell radioresistance. Folia Biol (Praha).

[35] Chen H, Tian M, Jin L, Jia H, Jin Y (2015). PUMA is invovled in ischemia/reperfusion-induced apoptosis of mouse cerebral astrocytes. Neuroscience..

[36] Li XQ, Yu Q, Chen FS, Tan WF, Zhang ZL, Ma H (2018). Inhibiting aberrant p53-PUMA feedback loop activation attenuates ischaemia reperfusion-induced neuroapoptosis and neuroinflammation in rats by downregulating caspase 3 and the NF-κB cytokine pathway. J Neuroinflammation.

[37] Arbo BD, Bennetti F, Ribeiro MF (2016). Astrocytes as a target for neuroprotection: modulation by progesterone and dehydroepiandrosterone. Prog Neurobiol.

[38] Liu Z, Chopp M (2016). Astrocytes, therapeutic targets for neuroprotection and neurorestoration in ischemic stroke. Prog Neurobiol.

[39] Pöyhönen S, Er S, Domanskyi A, Airavaara M (2019). Effects of neurotrophic factors in glial cells in the central nervous system: expression and properties in neurodegeneration and injury. Front Physiol.

[40] Boucher TJ, McMahon SB (2001). Neurotrophic factors and neuropathic pain. Curr Opin Pharmacol.

[41] Bennett DL, Boucher TJ, Michael GJ, Popat RJ, Malcangio M, Averill SA (2006). Artemin has potent neurotrophic actions on injured C-fibres. J Peripher Nerv Syst.

[42] Luo Y, Zhou Y, Xiao W, Liang Z, Dai J, Weng X (2015). Interleukin-33 ameliorates ischemic brain injury in experimental stroke through promoting Th2 response and suppressing Th17 response. Brain Res.

[43] Yang Y, Liu H, Zhang H, Ye Q, Wang J, Yang B (2017). ST2/IL-33-dependent microglial response limits acute ischemic brain injury. J Neurosci.

