# Supplementary material for: Neuroprotective effect of astrocyte-derived IL-33 in neonatal hypoxic-ischemic brain injury
Source: J Neuroinflammation. 2020 Aug 28;17:251. doi: 10.1186/s12974-020-01932-z (PMC7455908; doi:10.1186/s12974-020-01932-z)
Supplement: Supplementary file 1 — Additional file 1: Supplementary Fig. S1. mRNA expression of neurotrophic factors GDNF and PSPN in HI brain tissue of neonatal mice with or without IL-33 treatment for 3 days. Data are mean ± SEM (n = 3 in each group). *P < 0.05 compared to untreated controls [file 12974_2020_1932_MOESM1_ESM.zip › Supplementary Fig S1 legend.docx]

**Fig. S1.** mRNA expression of neurotrophic factors GDNF and PSPN in HI brain tissue of neonatal mice with or without IL-33 treatment for 3 d. Data are mean ± SEM (n = 3 in each group). * *P* < 0.05 compared to untreated controls.
